# Convolutional neural networks for reconstruction of undersampled optical projection tomography data applied to in vivo imaging of zebrafish

**DOI:** 10.1002/jbio.201900128

**Published:** 2019-08-29

**Authors:** Samuel P. X. Davis, Sunil Kumar, Yuriy Alexandrov, Ajay Bhargava, Gabriela da Silva Xavier, Guy A. Rutter, Paul Frankel, Erik Sahai, Seth Flaxman, Paul M. W. French, James McGinty

**Affiliations:** ^1^ Department of Physics Imperial College London London UK; ^2^ The Francis Crick Institute London UK; ^3^ Department of Medicine Imperial College London London UK; ^4^ Institute of Metabolism and Systems Research University of Birmingham Birmingham UK; ^5^ Division of Medicine University College London London UK; ^6^ Department of Mathematics and Data Science Institute Imperial College London London UK

**Keywords:** neural networks, optical tomography, preclinical imaging

## Abstract

Optical projection tomography (OPT) is a 3D mesoscopic imaging modality that can utilize absorption or fluorescence contrast. 3D images can be rapidly reconstructed from tomographic data sets sampled with sufficient numbers of projection angles using the Radon transform, as is typically implemented with optically cleared samples of the mm‐to‐cm scale. For in vivo imaging, considerations of phototoxicity and the need to maintain animals under anesthesia typically preclude the acquisition of OPT data at a sufficient number of angles to avoid artifacts in the reconstructed images. For sparse samples, this can be addressed with iterative algorithms to reconstruct 3D images from undersampled OPT data, but the data processing times present a significant challenge for studies imaging multiple animals. We show here that convolutional neural networks (CNN) can be used in place of iterative algorithms to remove artifacts—reducing processing time for an undersampled in vivo zebrafish dataset from 77 to 15 minutes. We also show that using CNN produces reconstructions of equivalent quality to compressed sensing with 40% fewer projections. We further show that diverse training data classes, for example, ex vivo mouse tissue data, can be used for CNN‐based reconstructions of OPT data of other species including live zebrafish.

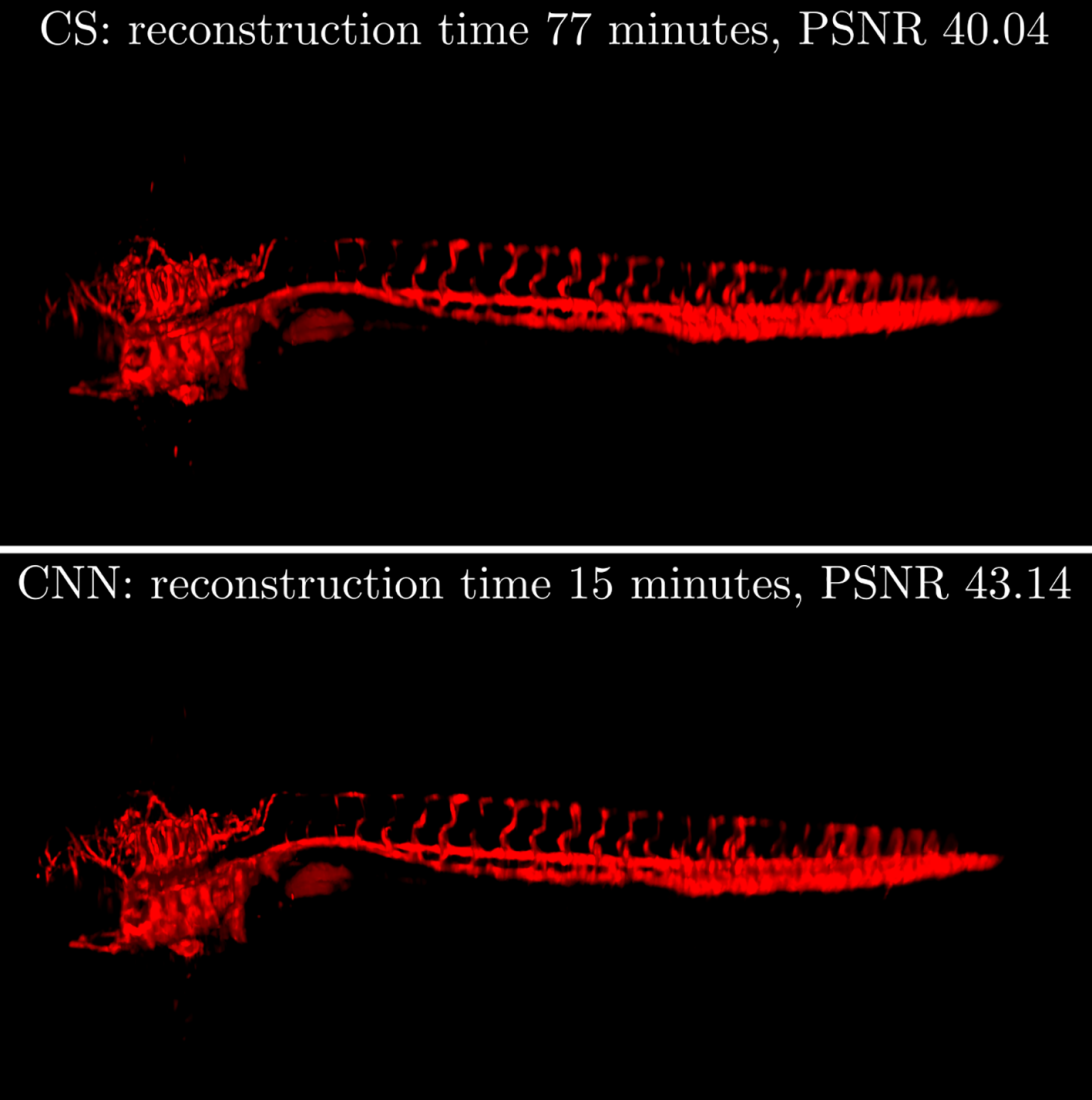

## Graphical abstract

Optical projection tomography is typically used for 3D imaging of fixed cleared tissue or whole organisms. in vivo applications require faster imaging, which is realized by acquiring less image data and reconstructing the tomographic images using iterative compressive sensing algorithms. However, these algorithms can be prohibitively slow for systematic studies. Instead, a neural network can reconstruct images much faster, with improved quality, and can be trained on ex vivo data.

## 1 INTRODUCTION

Optical imaging is now ubiquitous in biomedical research, extensively applied to planar samples such as two‐dimensional (2D) cell cultures and tissue sections and increasingly applied to extended tissue volumes and whole organisms. There is particular interest in three‐dimensional (3D) imaging in vivo, to provide a more realistic context 1 for the biology under study. Longitudinal imaging is highly desirable to mitigate against biological sample variability and to reduce animal numbers used in research.

Optical projection tomography (OPT) 2 is an established technique for 3D imaging in non/weakly scattering samples. Often described as the optical equivalent of X‐ray computerized tomography (CT), OPT entails the acquisition of wide‐field 2D transmission and/or fluorescence images of a rotating sample. It is frequently applied to mesoscopic sized volumes on the mm‐to‐cm scale, for example, to phenotype chemically cleared small animal organs 3, 4, 5, 6, and the low phototoxicity associated with wide‐field imaging makes it suitable for extended in vivo imaging studies, for example, of zebrafish embryos 7 and adult zebrafish 8—the latter mutated 9, 10 to suppress melanin and iridophore production.

For ex vivo samples, images are acquired at hundreds of different projection angles, from which the 3D volume can be analytically calculated using filtered back projection (FBP) 11, which typically takes a few minutes on a modest graphical processing unit (GPU)‐enabled personal computer. For in vivo OPT, however, it is highly desirable to reduce the length of time an animal is anesthetized, thereby minimizing side‐effects and possible confounding factors linked to anesthesia, and reducing the likelihood of unwanted movement during the OPT data acquisition 12. In addition, it is also important to minimize the light dose (and therefore the total OPT data acquisition time) to minimize phototoxicity or photobleaching of fluorescent proteins. While improvements in light collection efficiency, and therefore reductions in OPT acquisition times, have been realized by increasing the imaging numerical aperture, for example, through angularly‐multiplexed acquisition 13 or focal‐scanning OPT 14, 15, more significant reductions in total acquisition time can realized by reducing the number of projection images acquired. This also decreases the data volume and therefore data storage requirements.

Unfortunately, reducing the number of angular projections in a computed tomography data set significantly below the number set by the Shannon‐Nyquist sampling theorem leads to streak artifacts in images reconstructed using FBP 11. This impact of undersampled OPT data sets can be mitigated using compressed sensing (CS) algorithms 16 to significantly reduce the number of projections required to reconstruct faithful 3D images for suitable sparse samples. Unfortunately, due to the iterative nature of these reconstruction algorithms, which require multiple rounds of regularization and both forward‐projection and back‐projection per reconstructed slice, CS introduces a significant computational cost. In our laboratory, for example, a typical OPT data set that would normally take a few minutes to reconstruct using FBP implemented on a GPU‐accelerated desktop computer would require more than an hour to reconstruct using the two‐step iterative shrinkage/thresholding (TwIST) CS algorithm 17. This therefore introduces a data processing bottleneck for highly sampled longitudinal or high throughput studies.

Convolutional neural networks (CNN) provide an alternative computational approach for this and many other image data processing applications 18. In X‐ray CT, a U‐net CNN has been used to remove the streak artifacts from FBP reconstructions calculated from 7 and 20 times undersampled data 19. While the theoretical basis of this approach to deep learning is still being developed, for example 20, it provides an empirically useful approach to accelerating reconstruction of sparse tomography data. In this paper we report on CNNs applied to provide streak artifact‐free reconstructions of undersampled OPT data that have been trained using high quality 3D images of chemically cleared tissue volumes reconstructed using FBP of densely sampled OPT data. This CNN approach enables us to reconstruct undersampled OPT data five times faster compared to our previous iterative CS‐OPT approach and we demonstrate that CNN reconstructions can provide equivalent image quality to CS with 40% fewer projections, thereby further reducing the OPT data acquisition time. For in vivo applications, we show that a CNN trained on ex vivo immunostained mouse tissue OPT data can also be applied to reconstruct in vivo OPT datasets of zebrafish embryos expressing fluorescent proteins. This demonstrates the ability of the method to work across different types of biological sample and expands the scope of training data that can be used. The latter point is a particular advantage with live imaging, where the acquisition of sufficient high quality and fully sampled training data from live zebrafish would be technically challenging and undesirable in terms of the number of animals required.

## 2 METHODS

### 2.1 Reconstruction of OPT data using FBP and CS

**Figure 1:**
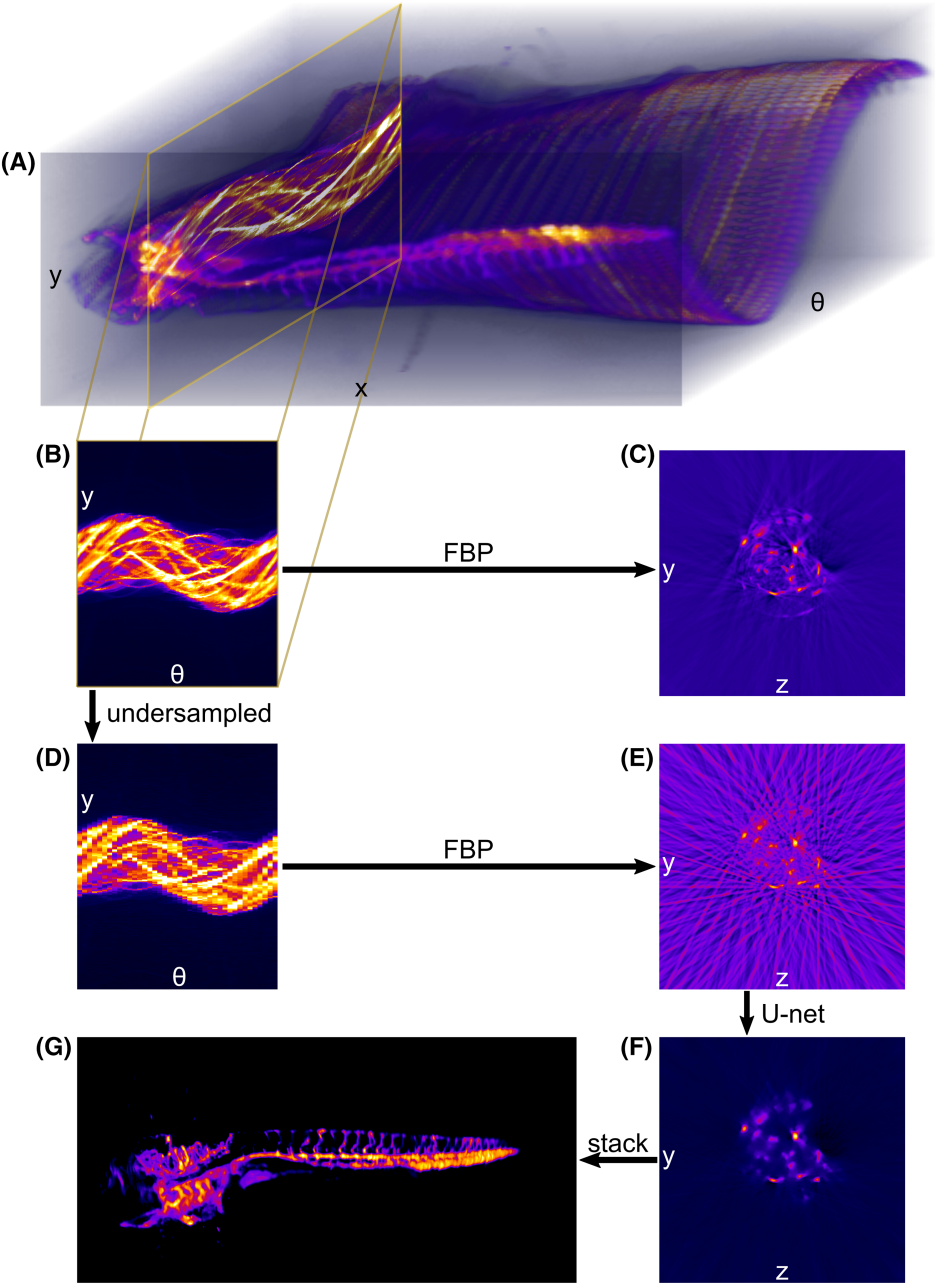
Convolutional neural networks for reconstructing undersampled optical projection tomography (OPT) data‐sets: A, represents an OPT dataset of a 4 days post fertilization zebrafish embryo, consisting of a series of 2D (x‐y) projections acquired as the sample is rotated to an angle θ; Each (y‐θ) cross section through the dataset forms a sinogram, (B, D), which can be processed with filtered back projection (FBP) to give a (y‐z) reconstructed slice, (C, E), of the sample volume. B and C, show the reconstruction of a highly sampled, 800 projection, dataset and (D, E) show the result of reconstructing from an undersampled, 40 projection, sinogram—resulting in a streak‐corrupted slice. F, shows the reconstruction obtained using a U‐net deep learning architecture—consisting of convolutional layers, max‐pooling, bilinear interpolation upscaling, and concatenated skip paths—to estimate the well‐sampled reconstruction from the undersampled FBP reconstruction (E). G, image obtained when the 2D reconstructed slices are stacked together to give the 3D fluorescence distribution of the sample

If the sample is contained within the depth of focus of a telecentric imaging system and the object presents negligible optical scattering, OPT data can be analyzed with parallel projection in the same way as X‐ray CT data. Thus, the forward model is given by:

(1) Y=RX+n

where *Y* is the measured 2D sinogram, *R* the Radon transform, *X* is the 2D fluorescence distribution of a slice of a 3D sample (Figure 1A‐C), and *n* is the measurement noise. For a well‐sampled sinogram *Y*, *X* can be analytically determined using FBP 11 (Figure 1B,C), which we have implemented in MATLAB using the built in *iradon* function.

For undersampled data, *X* can be recovered by solving the optimization problem:

(2) $$\min_{X}\frac{1}{2}{\left\|Y-\mathrm{RX}\right\|}_{2}^{2}+\tau \Phi_{\mathrm{TV}}\left(X\right)$$

where the first term is the *l*2‐norm of the residuals, τ is the regularization hyperparameter and Φ_*TV*_(*X*) is the total variation functional. To solve the problem represented by Equation (2) we employed a MATLAB implementation of TwIST 17, based on that demonstrated in 21. For the analysis presented in this paper, τ was kept constant at 0.004 for all reconstructions.

### 2.2 Convolutional neural network architecture to reconstruct undersampled OPT data

**Figure 2:**
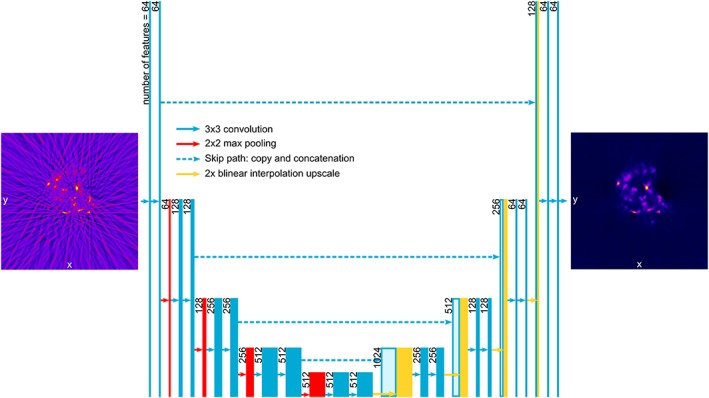
U‐net architecture convolutional neural network (CNN), which takes streak corrupted slices reconstructed from angularly undersampled optical projection tomography (OPT) datasets as inputs and outputs estimates of streak‐free slices reconstructed from well sampled datasets

To reconstruct undersampled OPT data (Figure 1D), CNNs with a modified U‐net architecture 22—implemented using the Python package PyTorch (https://pytorch.org/)—were trained to output an estimate for the well‐sampled (Figure 1C) reconstructed slice from the streak‐corrupted FBP reconstruction of under‐sampled OPT data (Figure 1E). The U‐net architecture (Figure 2) we implemented consisted of a contracting path of four blocks, each composed of two 3 × 3 convolutional layers followed by 2 × 2 max pooling (i.e., down‐sampling by taking the maximum local pixel value). After each max pooling step, the number of features doubled, starting from 64. The expanding path was also composed of four blocks, each up‐scaling the features by a factor of two. Due to GPU memory limits, this up‐scaling was performed using bilinear interpolation rather than with transpose convolutional layers. The expanded features were concatenated with those from the equivalent scale on the contracting path. The concatenated features were passed through two 3 × 3 convolutional layers and have their number quartered. Because transposed convolutional layers were not used, the number of features on the expanding path were half what they would be in a conventional U‐net. Zero padding was used in the convolutional layers to keep image sizes consistent between contracting and expanding paths.

Batch normalization layers were used after each convolutional layer to minimize internal covariate shift. These were followed by rectified linear unit layers to enable the network to account for possible nonlinear effects.

The CNNs were trained on 400‐projection OPT data‐sets from previous studies of ex vivo cleared mouse pancreas for which OPT was used to assay beta cell mass 23 and unpublished OPT studies of mouse lung. Eighty percent of the sinograms (14 416) were randomly assigned to training with the remaining 20% (3342) reserved for testing. These were assigned in batches of 10 neighboring slices to reduce correlations between testing and training data from adjacent sinograms.

CNNs were trained to remove streaks from slices reconstructed for a range of degrees of undersampling; 12, 16, 20, 24, 28, 32, 40, 48, 64 and 80 projections were used. Ground truth data (Figure 1C), *I*, were calculated using FBP of the complete 400‐projection sinogram data set (Figure 1B), with an additional Hann window applied to the ramp filter, scaled to 0.4. The input data, *I*_0_, consisted of FBP reconstructions from a reduced number of projections spaced as equally as the subsampling allowed (eg, a 64 projection sinogram used the 1st, 7th, 14th, 20th, 26th, etc. within the 400 projections). To augment the training data, a random angle in each sinogram was selected as the starting point (i.e., labeled 0°) and random vertical and/or horizontal flips were applied. Both ground truth and input FBP reconstructed slice pairs were normalized such that the input had a mean of zero and unit variance, and during training were randomly cropped to 128 × 128 pixels to transfer a batch size of 32 into GPU memory.

The CNN (Figure 2) takes the streak‐corrupted undersampled FBP reconstruction (Figure 1E) as its input and outputs an estimate for the streak‐free slice, $\hat{I}$ (Figure 1F). The network was trained using the error between $\hat{I}$ and *I* using the *l*1‐norm:

(3) $$l1=\frac{1}{n}\sum_{i=1}^{n}\left|{\hat{I}}_{i}-I_{i}\right|$$

where *i* is the pixel index. The *l*1‐norm was averaged over each batch of 32 slices and back‐propagated through the network to update the neuron weights. The Adam algorithm for stochastic gradient descent was used with the default parameters in PyTorch. We employed early stopping: after each pass of the training data, the average error on the test data was calculated and the training terminated when the error did not improve compared to the previous two epochs—the previous best network was kept. Training typically took between 3 and 6 hours on a desktop computer equipped with a Nvidia Tesla K40c, with 12 GB of memory. The training and testing scripts can be found online 24.

### 2.3 OPT data acquisition and samples

Fluorescence OPT of cleared mouse tissue samples was undertaken using a 0.5× telecentric lens (63‐074; Edmund Optics Ltd, Barrington, New Jersey) with a depth of field of 5 mm, imaging onto a charge‐coupled device camera (Retiga R1; QImaging, Surrey, Canada) for the pancreas samples and a 1× telecentric lens (58‐430; Edmund Optics Ltd) with a 5 mm depth of field, imaging onto a sCMOS camera (Zyla 5.5; Andor Instruments, Belfast, England) for the lung samples. Samples were suspended from a stepper motor (NM08AS‐T4‐MC04‐HSM8 and X‐MCB1‐KX11BG; Laser 2000 (UK) Ltd, Huntingdon, England) in a cuvette filled with index matching fluid and 400 equally spaced projections were acquired over a full rotation.

A total of nine pancreas samples were prepared following the protocol as described in 25. Briefly, the beta‐cell mass of whole fixed mouse pancreata were labeled with Alexa647‐conjugated antibodies. They were mounted in 2% agarose, dehydrated in increasing concentrations of methanol up to 100% and subsequently chemically cleared in a 1:2 mixture of benzyl alcohol: benzyl benzoate. Fluorescence excitation was provided by a 660 nm light‐emitting diode (Cairn Research Ltd, Faversham, England) in combination with a 705 ± 15 nm emission filter. A total of four lung samples were prepared. Briefly, KRAS^G12D^ TP53^frt/frt^ murine lung adenocarcinoma cells bearing yPET fluorescent protein were injected into the tail vain of ROSA26‐dTomato C57BL/6 mice (under authority of PPL70/8380). After 21–28 days, or at the clinically applicable humane endpoint, mice were sacrificed, and trachea were perfused with 2% low melting point agarose. Following this, lungs were placed in 4% PFA then 30% sucrose to allow lung specimen to retain fluorescence. Lastly, 30% sucrose was exchanged with Rapiclear RD: 1.52 to optically clear the tissue.

Fluorescence OPT of live zebrafish embryos was undertaken using a home‐built x4 microscope (N4X‐PF objective lens and ITL200 tube lens; Thorlabs Inc., Newton, New Jersey) with an aperture positioned directly behind the microscope objective lens to set the depth of field to 0.6 mm. The zebrafish embryo was suspended in a water filled cuvette from a stepper motor (T‐NM17A200; Zaber Technologies Inc., Vancouver, Canada) and images were recorded on a sCMOS camera (Zyla 5.5; Andor Instruments).

The zebrafish embryos (detailed description in Ref. 21) were derived from a mutant TraNac background to suppress melanin production (a gift from Julian Lewis, Cancer Research UK, London Research Institute, London, England) and were subsequently genetically modified to express mCherry fluorescence protein (mCherryFP) in the vasculature. mCherryFP fluorescence was excited using a 561 nm laser (Jive; Cobolt AB, Solna, Sweden) and imaged through a 641 ± 37 nm emission filter. The zebrafish embryos were imaged 4 days post‐fertilization, being anesthetized and mounted in fluorinated ethylene tubing with a refractive index that matched that of water.

## 3 RESULTS

### 3.1 Reconstruction of ex vivo mouse pancreas and lung OPT data

**Figure 3:**
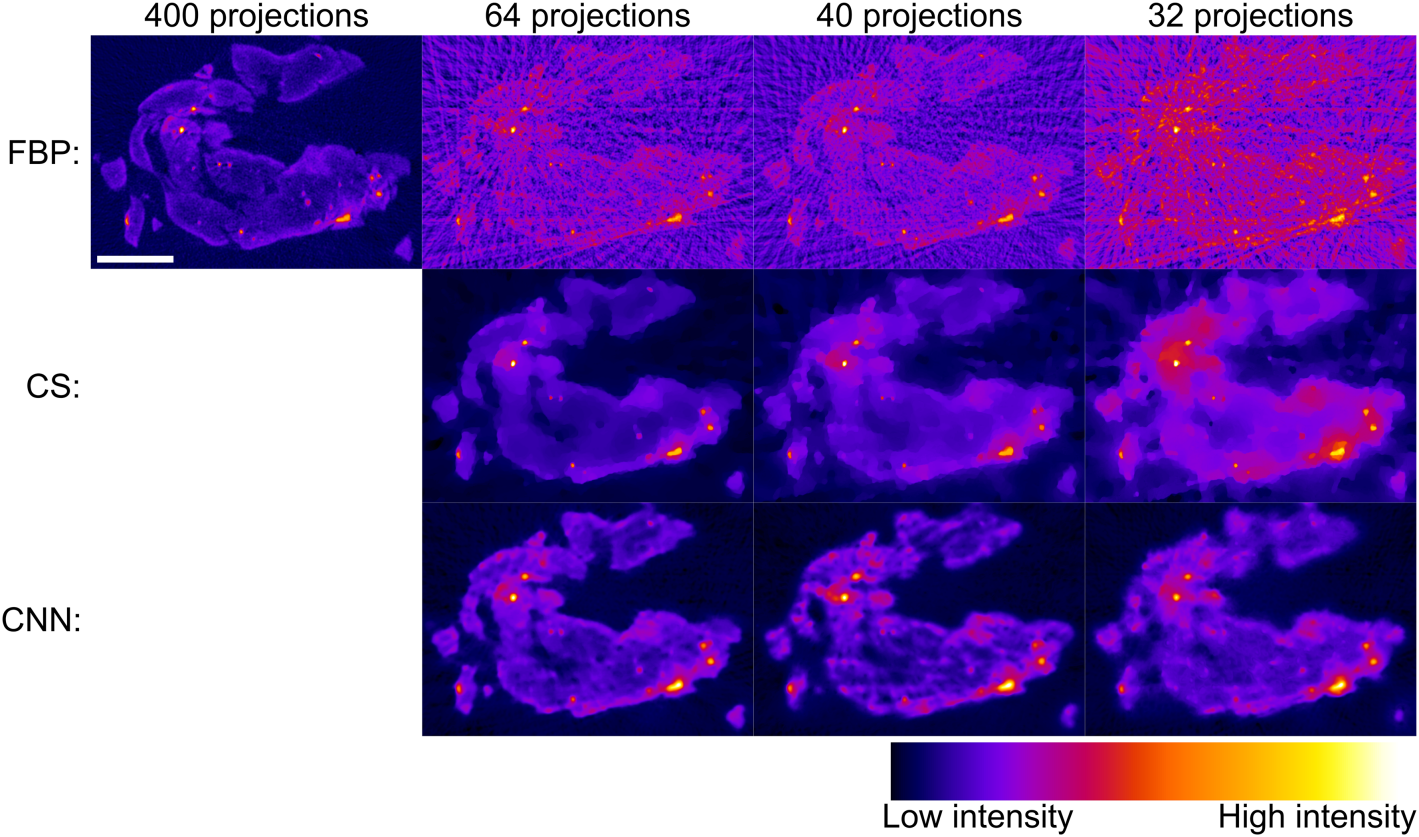
Comparison of optical projection tomography (OPT) reconstructions with false color intensity scale of a representative pancreas slice using filtered back projection (FBP), compressed sensing (CS), and convolutional neural networks (CNN), with different numbers of projections: compared to simple FBP of undersampled OPT data, CS and CNN methods are both able to provide significantly improved reconstructions. The CNN OPT reconstructions present reduced streak artifacts in the background compared to CS. Scalebar is 2 mm

Figure 3 shows representative image slices from the mouse pancreas OPT data reconstructed with a decreasing fraction of the 400 acquired projections and processed using either FBP, CS or CNN. As expected, the image quality of the FBP reconstructions degrades significantly as the number of projections is decreased. Streak artifacts become apparent across the whole of the reconstructed slice and the image contrast decreases. For modest undersampling with 64 projections, the iterative CS approach provides a visually improved reconstruction with significantly reduced streak artifacts and improved contrast, although the CS algorithm also smooths the reconstruction, leading to a slight reduction in apparent resolution. The CS reconstructions degrade further with increased undersampling: artifacts appear in background regions of the reconstructed images and the contrast is further reduced. The CNN approach outperforms both the FBP and CS reconstructions of undersampled OPT data, showing significantly reduced streak artifacts and higher image contrast.

**Figure 4:**
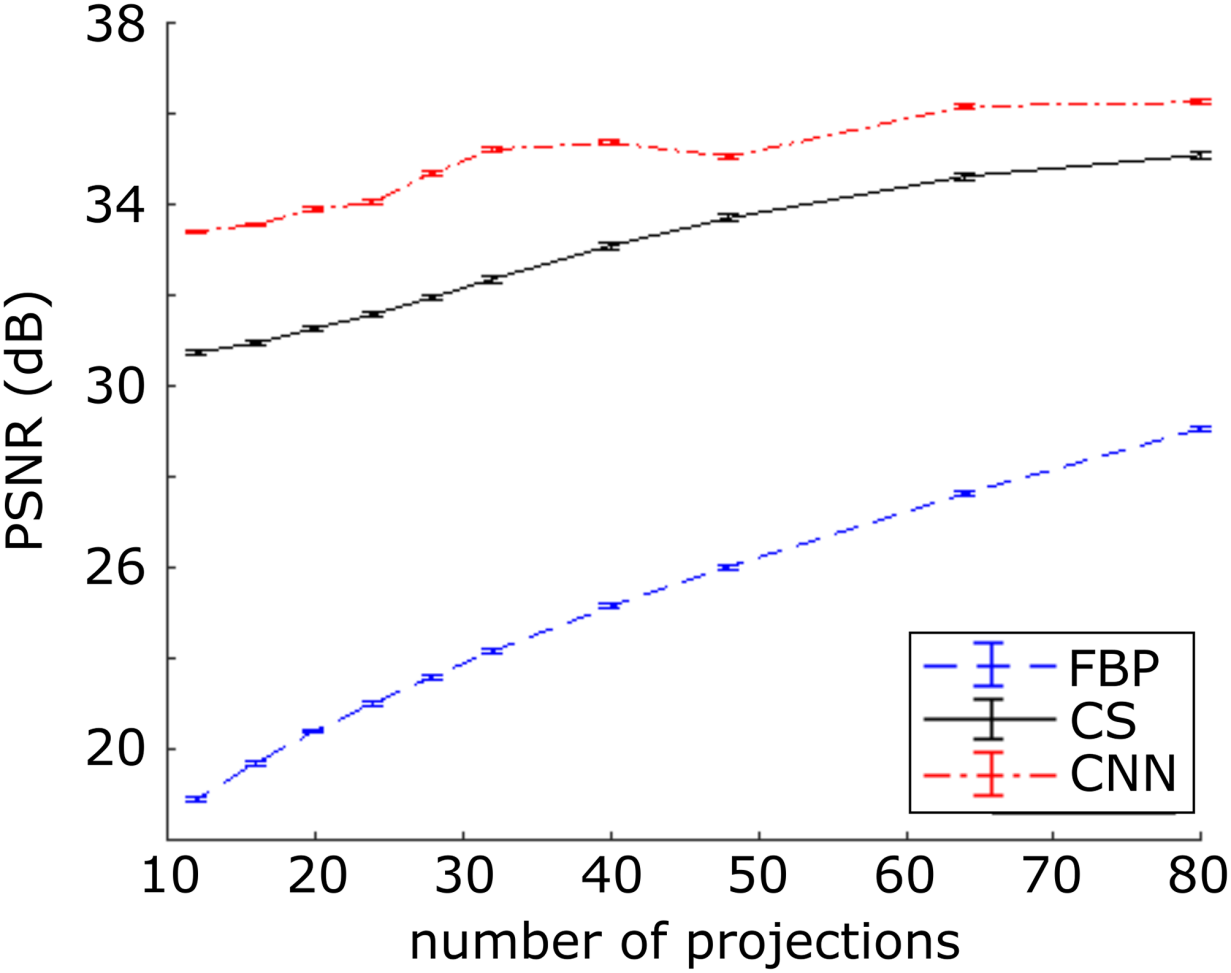
Variation of ratio of peak signal to noise ratio (PSNR) between undersampled optical projection tomography (OPT) reconstructions and “ground truth” for pancreas and lung test data‐set. The graph plots mean and standard error of measurements across the test data‐set of 3342 slices

The performance of the three approaches reconstructing undersampled OPT data was quantitatively assessed by calculating the peak signal to noise ratio (PSNR) with respect to the “ground truth” reconstruction over 3342 pancreas and lung slices (i.e., FBP on OPT data with 400 projections), shown in Figure 4.

The PSNR, in dB, is given by:

(4) $$\text{PSNR}=10\log_{10}\frac{I_{\mathrm{MAX}}}{\frac{1}{n}\sum_{i=1}^{n}{\left\|{\hat{I}}_{i}-I_{i}\right\|}^{2}}$$

where *I*_MAX_ is the peak pixel value of *I*, and the denominator is the mean squared error between *I* and $\hat{I}$.

The PSNR for the FBP reconstructions is seen to decrease as fewer projections are used. The CS and CNN reconstructions provide superior reconstructions compared to FBP, with the PSNR decreasing more slowly with fewer projections. Moreover, the CNN approach consistently outperforms CS. Therefore, employing CNN for the reconstruction should enable the OPT data acquisition times to be reduced compared to CS for equivalent PSNR in the reconstructed images.

### 3.2 Reconstruction of in vivo zebrafish OPT data

A key requirement for implementing CNN‐based approaches to image processing is a sufficient quantity and quality of training data. For OPT of fixed and cleared tissue samples, such high‐quality data can be easily obtained by acquiring a large number (i.e., hundreds) of projection images since there is no restriction on total acquisition time beyond possible photobleaching of the fluorescent labels. Such data sets are already available from many previous OPT studies of ex vivo tissue.

The acquisition of high‐quality in vivo training OPT data is much more challenging, since the total acquisition time may be practically restricted. For example, in our previous in vivo OPT study of tumor and vascular development in adult zebrafish 8, the total acquisition time was limited to 15 minutes by the maximum time we could confidently maintain the fish under anesthesia and be able to recover them. This time was sufficient for two (spectrally distinct) OPT acquisitions of only 64 projections per acquisition. While this undersampled OPT data can be reconstructed using the CS approach, it is not suitable as a CNN training data set.

Fortunately, it is not necessary to acquire in vivo training data. One practical approach would be to chemically clear a number of zebrafish and image them ex vivo with suitably high numbers of projections. However, this would still require a significant number of zebrafish to be specifically sacrificed to gather sufficient training data for the CNN approach. Instead, we reasoned that, while the 3D structure of previously imaged ex vivo mouse tissue is significantly different compared to the labeled structures in the zebrafish, the reconstructed OPT slices are similarly sparse, with the fluorescence signal localized to multiple small regions (eg, the islets in the mouse pancreas and the vasculature in the zebrafish). Consequently, the artifacts produced by FBP applied to undersampled data should be similar. We therefore applied our CNNs that were trained on the undersampled mouse tissue volumes described above to reconstruct in vivo OPT data of a transparent zebrafish line expressing mCherryFP in the vasculature: Tg(kinase insert domain receptor:mCherryFP).

**Figure 5:**
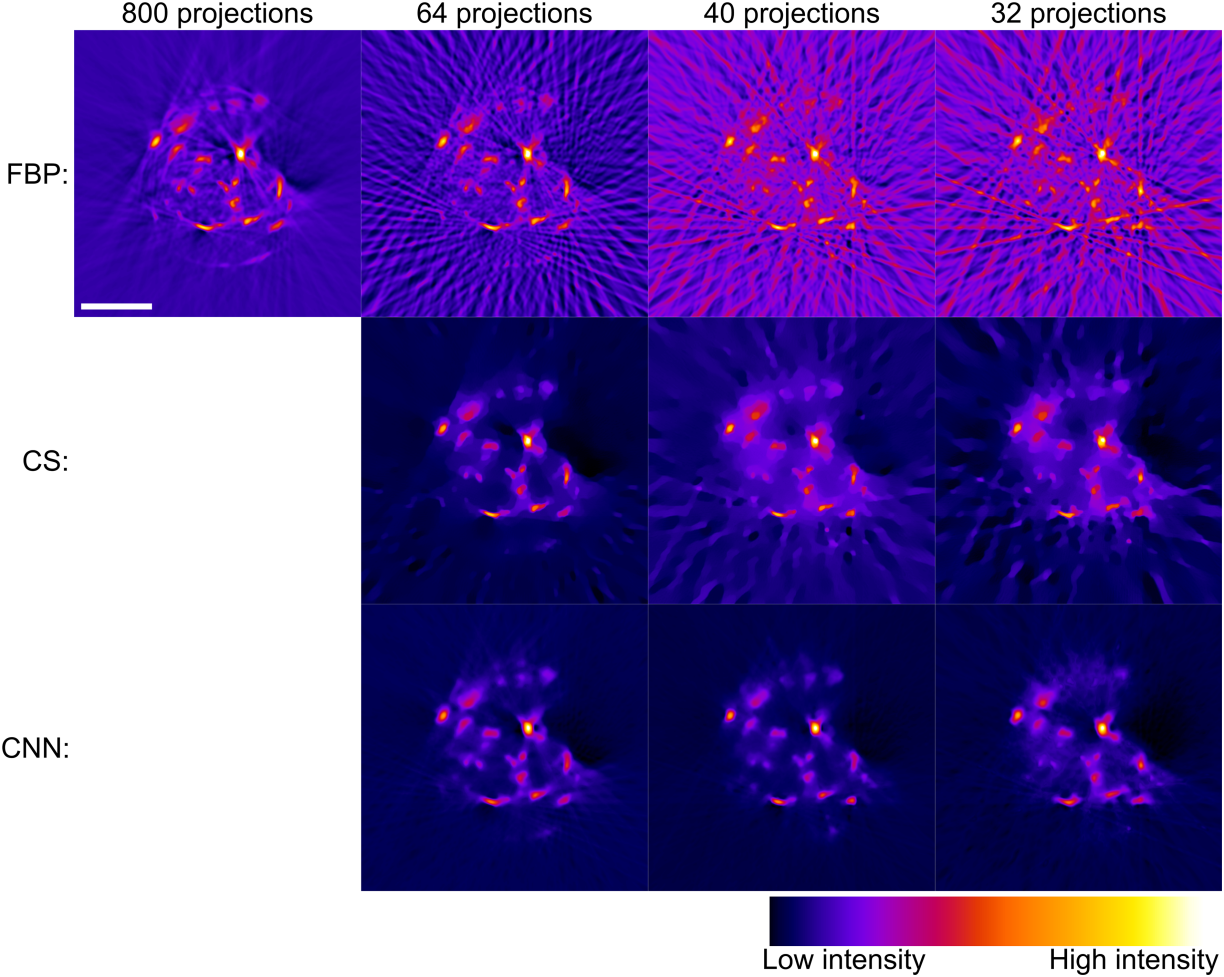
Comparison of optical projection tomography (OPT) reconstructions with false color intensity scale of a zebrafish embryo slice using filtered back projection (FBP), compressed sensing (CS), and convolutional neural networks (CNN) with different numbers of projections. The CS and CNN approaches produce acceptable reconstructions with significantly fewer projections than FBP. Below 64 projections, CS reconstructions are partially corrupted with streaks, while the CNN approach produces acceptable reconstructions using only 40 projections. Scalebar: 200 μm

Figure 5 shows a comparison of OPT image slices from an in vivo zebrafish embryo OPT data set from the study reported in 21 reconstructed using the FBP, CS and CNN approaches for a decreasing fraction of 800 acquired projections. As observed with the reconstructed images of mouse tissue, the FBP data suffers from increasingly significant streak artifacts and decreased contrast as the number of projections is reduced. The CS approach provides a reasonable reconstruction with 64 projections but presents increasing streak artifacts and reduced image contrast with images reconstructed from 40 and 32 projections. In contrast, the images reconstructed using the CNN approach presents higher image quality with fewer apparent streak artifacts and better preservation of the localized vascular signal (Video S1 in Appendix S1, Supporting Information).

**Figure 6:**
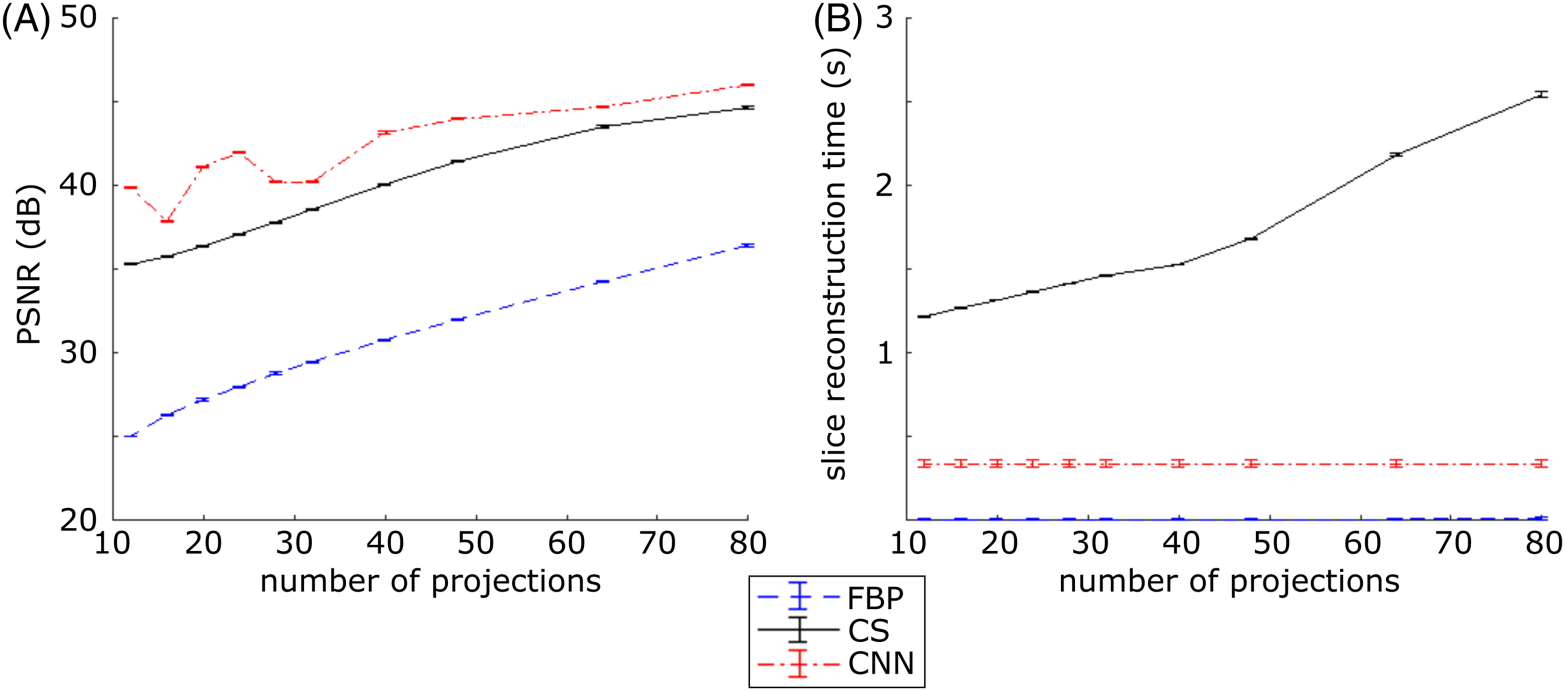
A, variation of peak signal to noise ratio (PSNR) of image slices of zebrafish embryo in vivo optical projection tomography (OPT) data between undersampled OPT reconstructions using filtered back projection (FBP), compressed sensing (CS), and convolutional neural networks (CNN) (trained on mouse tissue) and “ground truth” based on FBP of 800 projection images of zebrafish and (B) average OPT image reconstruction time per slice, using FBP, CS, and CNN. Graph shows mean and standard error of PSNR of 2218 reconstructed slices

Figure 6A shows how the PSNR between slices reconstructed from fully sampled and undersampled projection data, averaged over the 2218 slices of the zebrafish embryo, varies with degree of undersampling. As seen with the mouse tissue, the PSNR value improves from FBP to CS to CNN for reconstructions with the same number of projection images. The PSNR for the CS and CNN reconstruction decreases more significantly with undersampling than was observed with the mouse pancreas OPT data in Figure 4. However, the CNN approach still outperforms the FBP and CS approaches and employing CNN for the OPT image reconstruction achieves equivalent PSNR compared to a 64 projection CS reconstruction with 40 projections—enabling a reduction in data acquisition time of 40%. The PSNR measures the image reconstruction performance averaged across all reconstructions. To explore the dynamic range, an investigation of the reconstruction of specific features with different signal levels is included in supplementary information S1.

The decrease in processing time per slice for the CNN approach compared to CS is even more significant. Figure 6B shows the average data processing time per reconstructed slice for the FBP, CS and CNN approaches implemented on the same desktop computer with an Nvidia Tesla K40c GPU. We note that FBP is an established analytic reconstruction technique that has been optimized in MATLAB and required only (8.75 ± 0.01) × 10^−3^ seconds to reconstruct each slice from 64 projections. The CNN reconstruction approach takes FBP reconstructions as input and performs one feedforward pass through the neural network, so the additional processing time is independent of the number of projection images, with a value of (3.40 ± 0.02) × 10^−1^ seconds per slice for 64 projections. In contrast, the CS approach requires multiple rounds of forward‐ and back‐projection and regularization, resulting in a significantly increased overall reconstruction time per slice, which further increases with the number of projection images. The average reconstruction time per slice for our CS implementation was 2.18 ± 0.01 seconds for 64 projections. Thus, for reconstructions from OPT data with 64 projections, the iterative CS approach was 6× slower per slice compared to CNN, and its reconstruction quality, as indicated by the PSNR metric, was inferior. The total OPT image reconstruction time for the zebrafish data set comprising 2218 slices with 64 projections was 15 minutes for the CNN approach, compared to 77 minutes required for the CS approach. This includes common overheads such as the time required for loading and saving the data to disk.

## 4 DISCUSSION

OPT can offer accelerated imaging of sparse samples by utilizing compressive sensing techniques to reconstruct undersampled OPT data sets. This is essential for in vivo OPT and convenient whenever smaller data volumes are desired. Here we have extended previous work 21 to demonstrate the use of CNNs for significantly faster and improved reconstruction of undersampled OPT data compared to an iterative compressive sensing algorithm. We have developed this novel approach to OPT applied to data from previous studies of chemically cleared and labeled mouse tissue and have demonstrated that the resulting CNNs are also applicable to in vivo zebrafish OPT data. Significantly, the mouse tissue‐trained CNN maintain their relative reconstruction quality and speed advantage compared to iterative CS reconstruction despite no zebrafish data being included during the network training process. This is important in terms of reduced numbers of animals being sacrificed for in vivo research.

The improved image quality and data processing speed provided by the CNN approach to reconstructing undersampled OPT data will be important for longitudinal and high throughput 3D preclinical imaging studies of zebrafish and other small/transparent organisms, for which the substantial computational challenge presented by the iterative algorithm of the CS approach is a significant bottleneck that can delay or preclude important feedback to the ongoing study (eg, informing optimization of data quality or animal welfare).

## CONFLICT OF INTEREST

The authors have no competing interests to declare.

## AUTHOR CONTRIBUTIONS

S.P.X.D., P.M.W.F. and J.M. conceived and designed the study. G.A.R. and G.d.S.X. provided the mouse pancreas OPT data from a previous study [25]. E.S., A.B. and S.K. provided the mouse lung OPT data from experiments undertaken by A.B. and S.K. P.F., J.M. and P.M.W.F. provided the zebrafish data from a previous study [21] performed by J.M. S.P.X.D. designed the CNN supervised by S.F. and analyzed the OPT data supervised by P.M.W.F. and J.M. All authors contributed to and reviewed the manuscript.

## Supporting information


**Appendix S1**. Supplementary Information

**Figure S2**. A, Slice of zebrafish embryo reconstructed with filtered back projection (FBP) from a fully sampled (800 projections) dataset, with three regions of interest highlighted. Scalebar 250 μm. B, C, E, Regions of interest around features (*i, ii, iii)*, reconstructed with FBP, compressed sensing (CS) and convolutional neural networks (CNN) approaches for different numbers of angular projections. The feature (*i*) is resolved in all cases shown while the feature (*ii)* is resolved down to 48, and 40 projections for CS and CNN reconstructions respectively. D, Line profiles through *ii* as indicated in (C). The feature (*iii)* is not resolved in the CS reconstructions but is discernible in the 80 projection CNN reconstruction. F, Line profiles through feature (*iii)* as indicated in (E).

**Video S1.** Comparison of optical projection tomography (OPT) reconstructions with false color intensity scale of a zebrafish embryo using (A) filtered back projection (FBP), (B) compressed sensing (CS) and (C) convolutional neural networks (CNN), using 40 projections: compared to simple FBP of undersampled OPT data, CS and CNN methods are both able to provide significantly improved reconstructions. The CNN OPT reconstruction presents reduced streak artifacts in the background compared to CS. Scalebar is 250 μm.
