# Supplementary material for: Convolutional neural networks for reconstruction of undersampled optical projection tomography data applied to in vivo imaging of zebrafish
Source: J Biophotonics. 2019 Aug 29;12(12):e201900128. doi: 10.1002/jbio.201900128 (PMC7065643; doi:10.1002/jbio.201900128)
Supplement: Supplementary file 1 — Appendix S1. Supplementary Information [file JBIO-12-e201900128-s001.docx]

**Convolutional neural networks for reconstruction of undersampled optical projection tomography data applied to *in vivo* imaging of zebrafish**

**Supplementary information**

Samuel P. X. Davis^1^*, Sunil Kumar^1,2^, Yuriy Alexandrov^1,2^, Ajay Bhargava^2^, Gabriela da Silva Xavier^3,4^, Guy A. Rutter^3^, Paul Frankel^5^, Erik Sahai^2^, Seth Flaxman^6^, Paul M. W. French^1,2^† and James McGinty^1,2^†

^1^ Department of Physics, Imperial College London, London, SW7 2AZ, United Kingdom

^2^ The Francis Crick Institute, London, NW1 1AT, UK

^3^ Department of Medicine, Imperial College London, London SW7 2AZ, UK

^4^ Institute of Metabolism and Systems Research, University of Birmingham, Birmingham B15 2TT, UK

^5^ Division of Medicine, University College London, University Street, London WC1E 6JF, UK

^6^ Department of Mathematics and Data Science Institute, Imperial College London, London, SW7 2AZ, United Kingdom

*s.davis15@imperial.ac.uk

†These authors contributed equally to this work

Supplementary figure 1: Comparison of OPT reconstructions with false color intensity scale of a zebrafish embryo using (**a**) FBP, (**b**) CS, and (**c**) CNN, using 40 projections: compared to simple FBP of under-sampled OPT data, CS and CNN methods are both able to provide significantly improved reconstructions. The CNN OPT reconstruction presents reduced streak artifacts in the background compared to CS. Scalebar is 250 μm.

**Sensitivity of CS and CNN reconstructions**

Successful reconstruction of an object from undersampled data using CS techniques such as TwIST requires that the sample is sparse in some domain. In this experiment gradient sparsity is enforced, which can be seen in equation 2. In selecting a value for the hyperparameter $,$ a compromise must be made between specificity (how well streaks are rejected) and accuracy (how well the object features are preserved). By enforcing greater sparsity, more streaks are removed, but so are less prominent features present in the object. Similarly, less prominent features will produce smaller errors, and therefore take lower precedence than large features and the ubiquitous streak artifacts in the training of the CNN. Therefore, it can be expected that both CS and CNN methods should present worse performance recovering less prominent features.

Illustrating an attempt to explore the sensitivity of the reconstruction to small feature brightness, figure 7 shows regions of interest around several features of different brightness in a reconstructed slice of a zebrafish embryo. In the fully sampled FBP reconstruction, the intensities of the first, second, and third features are 53%, 25%, and 17% of the intensity of the brightest feature in the field of view. These regions of interest from the images reconstructed with FBP, CS, and CNN are shown for different numbers of angular projections. The brightest feature (i) is visible for all datasets shown. However, the second (ii) and third (iii) features eventually cease to be discernible as the number of projections in the dataset is reduced – but in both cases these features are resolvable in the CNN images for greater degrees of undersampling than for the CS reconstructions.


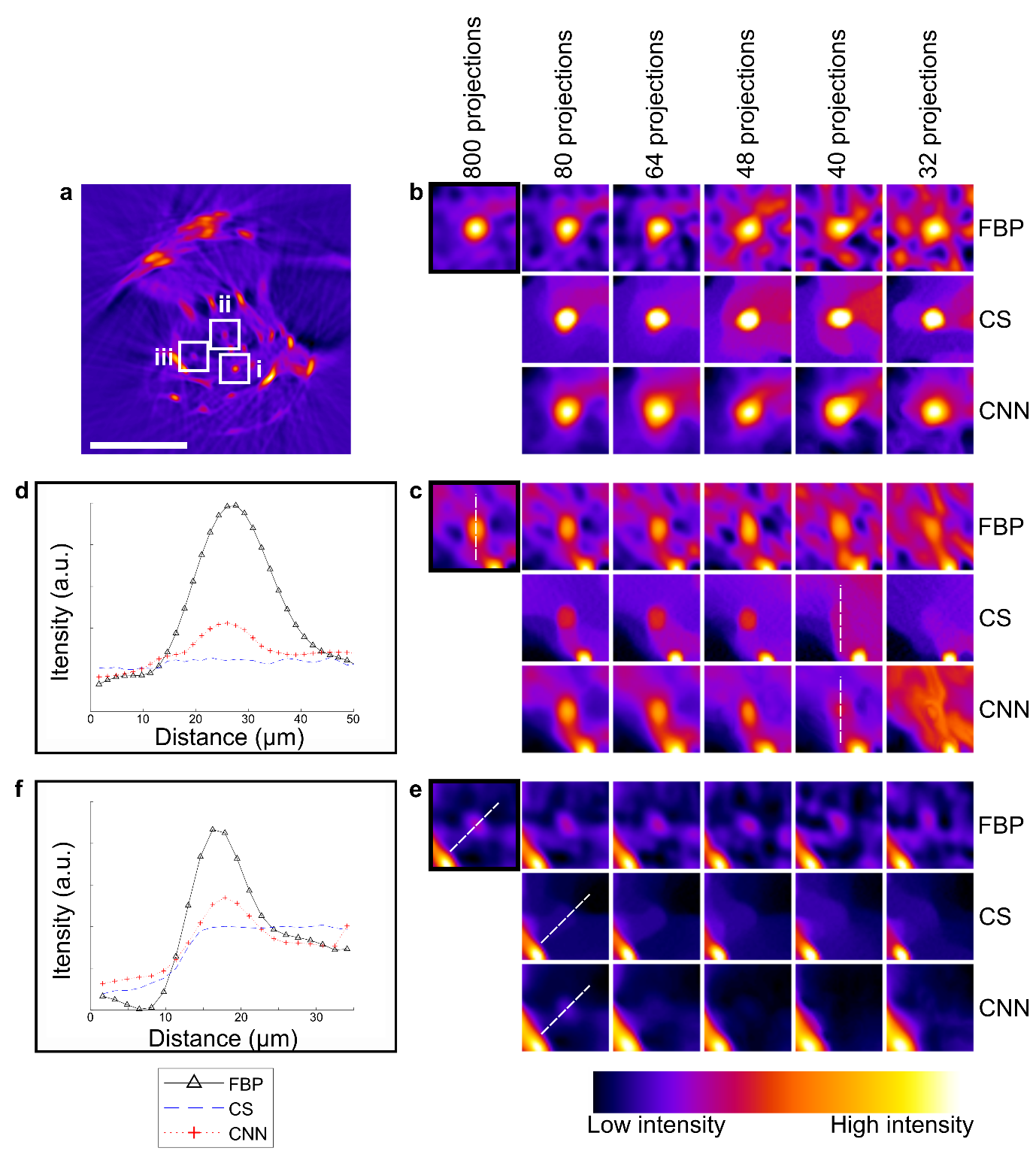


Supplementary figure 2: (a) Slice of zebrafish embryo reconstructed with FBP from a fully sampled (800 projections) dataset, with three regions of interest highlighted. Scalebar 250 μm. (b, c, e) Regions of interest around features (*i, ii. iii)*, reconstructed with FBP, CS and CNN approaches for different numbers of angular projections. The feature (*i*) is resolved in all cases shown while the feature (*ii)* is resolved down to 48, and 40 projections for CS and CNN reconstructions respectively. (d) Line profiles through *ii* as indicated in (c). The feature (*iii)* is not resolved in the CS reconstructions but is discernible in the 80 projection CNN reconstruction. (f) Line profiles through feature (*iii)* as indicated in (e).
