# Supplementary figures and images for: Convolutional neural networks for reconstruction of undersampled optical projection tomography data applied to in vivo imaging of zebrafish
Source: J Biophotonics. 2019 Aug 29;12(12):e201900128. doi: 10.1002/jbio.201900128 (PMC7065643; doi:10.1002/jbio.201900128)

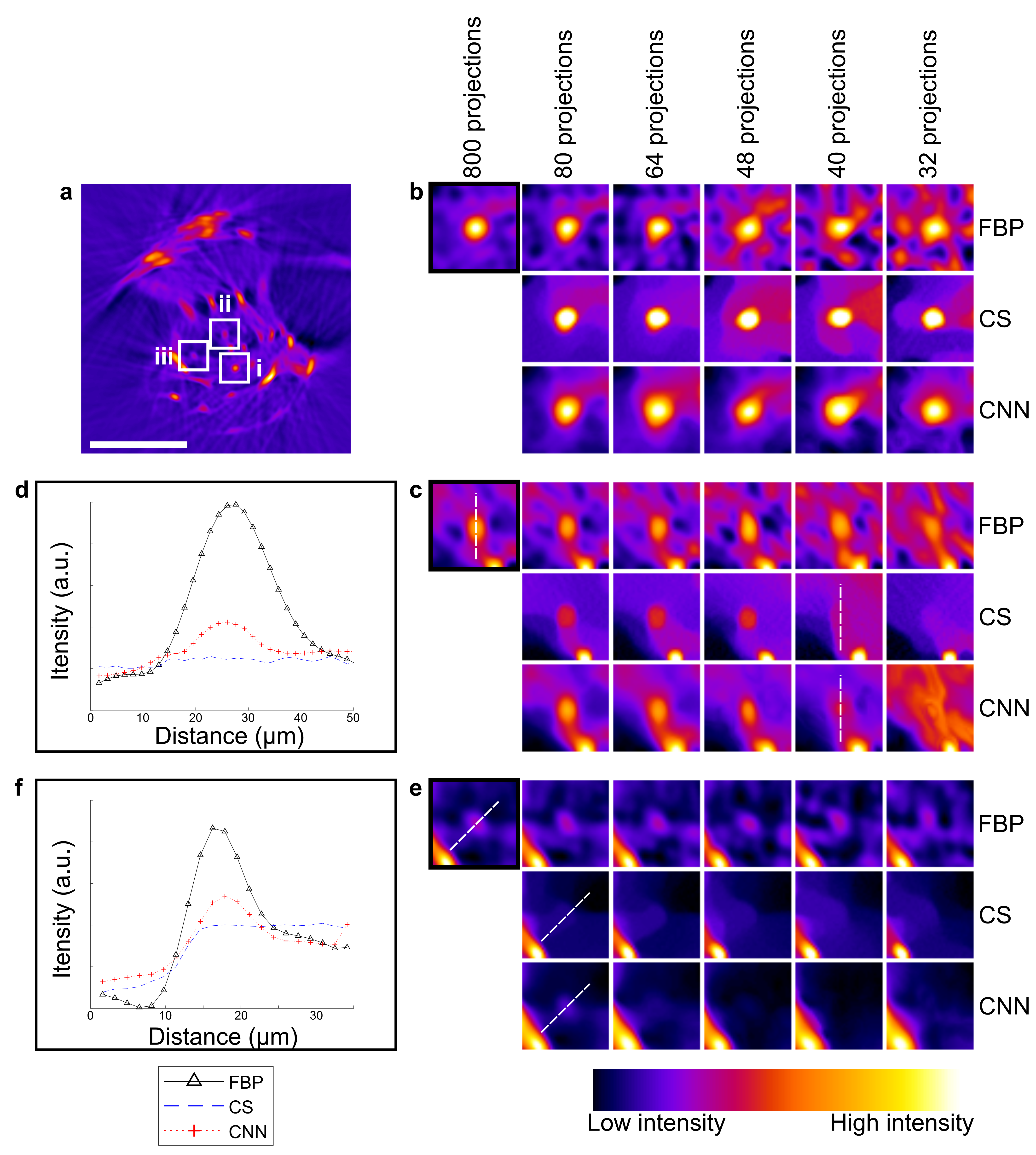

Supplement: Supplementary file 2 — Figure S2. A, Slice of zebrafish embryo reconstructed with filtered back projection (FBP) from a fully sampled (800 projections) dataset, with three regions of interest highlighted. Scalebar 250 μm. B, C, E, Regions of interest around features (i, ii, iii), reconstructed with FBP, compressed sensing (CS) and convolutional neural networks (CNN) approaches for different numbers of angular projections. The feature (i) is resolved in all cases shown while the feature (ii) is resolved down to 48, and 40 projections for CS and CNN reconstructions respectively. D, Line profiles through ii as indicated in (C). The feature (iii) is not resolved in the CS reconstructions but is discernible in the 80 projection CNN reconstruction. F, Line profiles through feature (iii) as indicated in (E). [file JBIO-12-e201900128-s002.png]

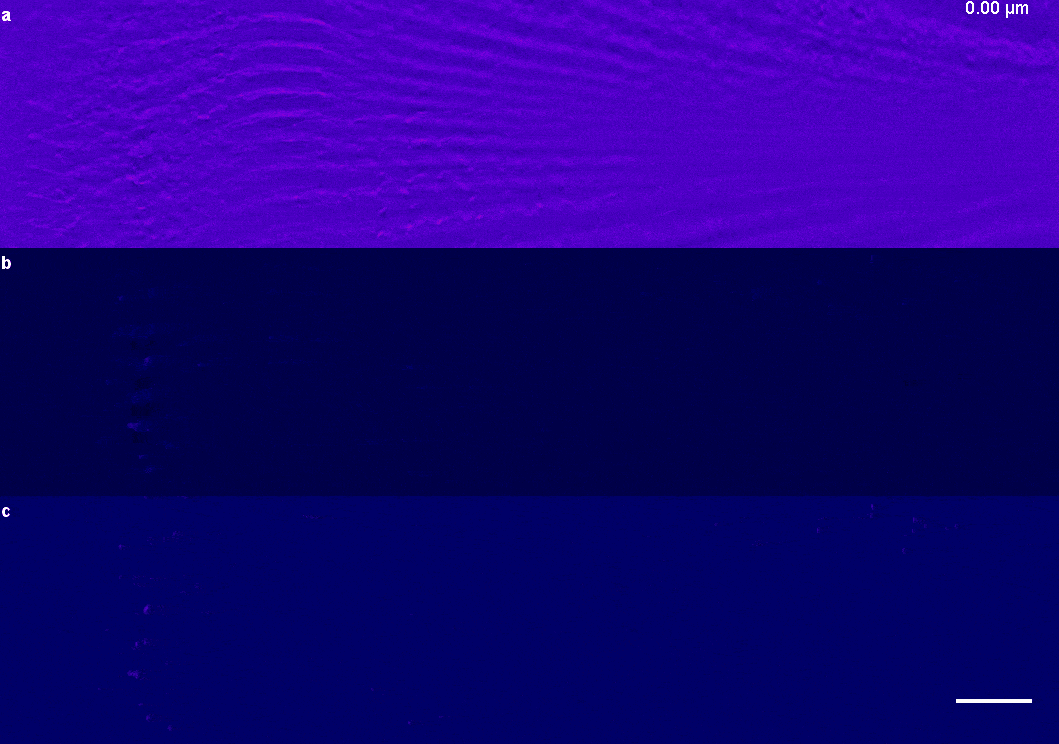

Supplement: Supplementary file 3 — Video S1. Comparison of optical projection tomography (OPT) reconstructions with false color intensity scale of a zebrafish embryo using (A) filtered back projection (FBP), (B) compressed sensing (CS) and (C) convolutional neural networks (CNN), using 40 projections: compared to simple FBP of undersampled OPT data, CS and CNN methods are both able to provide significantly improved reconstructions. The CNN OPT reconstruction presents reduced streak artifacts in the background compared to CS. Scalebar is 250 μm. [file JBIO-12-e201900128-s003.gif]
